# Beyond defense: GTR-mediated uptake of glucosinolates drives growth and structural reprogramming in broccoli seedlings

**DOI:** 10.1093/plphys/kiag556

**Published:** 2026-07-30

**Authors:** Lorena Albaladejo-Marico, Christa Kanstrup, Christoph Crocoll, Micaela Carvajal, Josip Tomas, Marie Schuler, Arnaud Tatibouet, Hussam Hassan Nour-Eldin, Lucia Yepes-Molina

**Affiliations:** Aquaporins Group, Centro de Edafologia y Biologia Aplicada del Segura, CEBAS-CSIC, Campus Universitario de Espinardo 25, Murcia E-30100, Spain; DynaMo Center, Department of Plant and Environmental Sciences, Faculty of Science, University of Copenhagen, Thorvaldsensvej 40, Frederiksberg C 1871, Denmark; DynaMo Center, Department of Plant and Environmental Sciences, Faculty of Science, University of Copenhagen, Thorvaldsensvej 40, Frederiksberg C 1871, Denmark; Aquaporins Group, Centro de Edafologia y Biologia Aplicada del Segura, CEBAS-CSIC, Campus Universitario de Espinardo 25, Murcia E-30100, Spain; GlycoBio&Chemistry Team, ICOA, UMR 7311, Université D’Orléans et CNRS, BP 6759, Orléans F-45067, France; GlycoBio&Chemistry Team, ICOA, UMR 7311, Université D’Orléans et CNRS, BP 6759, Orléans F-45067, France; GlycoBio&Chemistry Team, ICOA, UMR 7311, Université D’Orléans et CNRS, BP 6759, Orléans F-45067, France; DynaMo Center, Department of Plant and Environmental Sciences, Faculty of Science, University of Copenhagen, Thorvaldsensvej 40, Frederiksberg C 1871, Denmark; Aquaporins Group, Centro de Edafologia y Biologia Aplicada del Segura, CEBAS-CSIC, Campus Universitario de Espinardo 25, Murcia E-30100, Spain

## Abstract

Exogenous application of glucosinolates (GSLs) has previously been reported to act as a biostimulant, enhancing crop resilience and growth. However, the molecular mechanisms governing their uptake and metabolic integration remain unknown. In this study, we investigated the role of glucosinolate transporters (GTRs) in the active internalization of exogenous GSLs and their subsequent impact on the early development of broccoli (*Brassica oleracea* L. var. *italica*) seedlings. Using a multidisciplinary approach combining *Xenopus laevis* oocyte heterologous expression, confocal microscopy with fluorescent GSL derivatives, and RNA-seq analysis, we demonstrated that broccoli seedlings actively uptake exogenous GSLs through the root system. Accordingly, functional characterization confirmed that BolGTR1.2, BolGTR2.2, BolGTR2.3, and BolGTR3.3 are high-efficiency transporters capable of translocating both aliphatic and indolic GSLs. Together, GSL profiling, time-course RT-qPCR, and Day 7 RNA-seq revealed that GSL internalization triggers a biphasic response: an initial “consumption phase,” followed by a compensatory upregulation of de novo GSL biosynthetic machinery by Day 7. This metabolic “shortcut” bypasses the energetically costly sulfate reduction pathway, allowing for the reallocation of cellular energy toward structural growth, which was evidenced by the induction of cell wall remodeling genes (*XTH*, *PAE8*, *GAE*) and a measurable increase in seedling biomass. Collectively, our findings support the hypothesis that GSLs are not merely defensive compounds but dynamic metabolic reserves that coordinate resource allocation.

## Introduction

Glucosinolates (GSLs) are sulfur-rich secondary metabolites characteristic of the Brassicaceae family (Falk et al. 2007). They are well recognized for their ecological role in plant defense against herbivores and pathogens and for their involvement in regulating plant growth, development, and responses to environmental stress (Variyar et al. 2014). The structural diversity of GSLs is traditionally classified according to their amino acid precursors: aliphatic GSLs, mainly derived from methionine; indolic GSLs, derived from tryptophan; and phenylalanine- or tyrosine-derived aromatic GSLs (Sikorska-Zimny and Beneduce 2021). The Brassicaceae family exhibits the highest concentration and variability of these compounds (Bird et al. 2025), with significant intraspecific fluctuations in GSL profiles depending on species, plant tissue, and environment (Katz et al. 2021). Beyond their physiological functions in plants, GSLs and their hydrolysis products have attracted considerable interest due to their health-promoting properties in humans, including anticancer, antioxidant, and anti-inflammatory activities (Traka 2016). Consequently, research on these compounds has intensified in recent years, substantially expanding our understanding of their roles across multiple fields, including plant biotechnology.

Recently, evidence has emerged suggesting that GSLs may also act as biostimulants in plants. Previous work from our group demonstrated that exogenous application of the major aliphatic and indolic GSLs naturally present in broccoli (*Brassica oleracea* L. var. *italica*), namely, 4-(methylsulfinyl)butyl glucosinolate (4-MSB) and indol-3-ylmethyl glucosinolate (I3M), to broccoli seeds significantly increased seedling biomass (Albaladejo-Marico et al. 2024). These results suggest a potential role for specific GSLs in promoting early developmental processes. Accordingly, transcriptomic analysis revealed that a GSL-rich extract appears to redirect the energy resources of broccoli plants, shifting the transcriptional profile from standard stress responses toward growth-promoting pathways (Albaladejo-Marico et al. 2025a). Also, other previous works have shown that externally supplied GSLs can accumulate within plant tissues under specific experimental conditions, such as the accumulation of a non-endogenous GSL in *Arabidopsis thaliana* following root feeding (Andersen and Halkier 2014; Jeschke et al. 2019). However, it remains unclear how this internalization is mediated at the cellular level and how it contributes to early seedling establishment and growth responses.

The uptake and intracellular distribution of GSLs are thought to be mediated by specific transport proteins. Among these, the GSL transporters (GTRs), members of the NPF family of proton symporters, are the best characterized (Jørgensen et al. 2017a, 2017b). GSL transporters have been implicated in long-distance translocation of GSLs, their allocation between source and sink tissues, and their compartmentalization within plant cells (Nour-Eldin et al. 2012; Jørgensen et al. 2015; Xu et al. 2019). Crucially, GTRs display distinct substrate specificities depending on the core molecular structure and side-chain modifications of the GSL species (Figure S1), which dictate their transport affinity and efficiency (Jørgensen et al. 2017a, 2017b). Their substrate specificity and physiological relevance suggest they may represent key mediators of exogenous GSL uptake, yet their specific contribution has not been studied.

To address these questions at the mechanistic level, heterologous expression systems provide a powerful experimental framework. *Xenopus laevis* oocytes are widely used to characterize the activity of plant transporters due to their large size, ease of manipulation, and ability to faithfully translate exogenous cRNA (Bhatt et al. 2022). When expressed in oocytes, candidate GTR proteins can be directly tested for their capacity to mediate uptake of GSLs, offering mechanistic insights that complement *in planta* studies. In addition, the use of fluorescently labeled GSLs has recently emerged as a valuable approach for monitoring cellular uptake and subcellular localization, further advancing our ability to dissect GSL transport processes. Using this strategy, Kanstrup et al. (2023a) demonstrated that the *A. thaliana* GTR1/GTR2/GTR3 transporters are capable of mediating the uptake of various GSLs conjugated to different fluorophores, validating the utility of this approach for functional studies.

Despite increasing evidence for biostimulatory effects of exogenous GSLs, the mechanisms underlying their uptake and their contribution to early growth responses remain poorly understood. In this study, we combined in vitro seedling growth assays, GSL quantification, microscopy, and heterologous expression in *X. laevis* oocytes to examine the uptake and physiological impact of pure GSLs in broccoli. Furthermore, we employed a transcriptomic approach to provide a global expression landscape. Through RNA-Seq analysis, we aimed to identify the metabolic pathways, regulatory networks, and candidate transporters involved in GSL perception, uptake, and downstream signaling. To our knowledge, this is the first demonstration that broccoli seedlings are capable of absorbing exogenous GSLs from the medium and incorporating them into their tissues, suggesting that GTR transporters may serve as their active entry mechanism. By addressing both physiological and mechanistic aspects, our work contributes to a deeper understanding of the role of GSLs in plant development and highlights their potential applications in crop improvement and biostimulation strategies.

## Methods

### Seed growth in vitro

Broccoli seeds (*B. oleracea* L. var. *italica*) were surface-sterilized in a 1:1 solution of sodium hypochlorite (NaClO) and sterile ddH_2_O and subsequently sown in 48-well Petri plates containing 0.5 mL of 0.8% agar medium per well. Two pure GSLs were incorporated into the medium: 4-MSB (0.08 µM) and I3M (2.53 µM). These concentrations were selected based on our previous work, where they matched the levels naturally present in a broccoli-derived biostimulant extract and were demonstrated to stimulate broccoli seedling growth when applied individually as pure compounds (Albaladejo-Marico et al. 2024). Seedlings were grown in a controlled-environment chamber for 7 days under the following conditions: 25 °C during the light period and 20 °C during darkness, with a 16-h light/8-h dark photoperiod. Relative humidity was maintained at 50%, and photosynthetically active radiation (PAR) was set at 300 µE, provided by LEDs. The fresh weight (FW) and dry weight (DW) of the seedlings were determined on Day 7.

### Determination of GSL content in seedlings

Glucosinolate content in treated and control seedlings at Days 1, 3, 5, and 7 was extracted as desulfo-GSL as previously described in (Kliebenstein et al. 2001). For Days 1 and 3, whole seedlings were harvested, weighed, and ground in liquid nitrogen, whereas shoots and roots were collected separately at Days 5 and 7. The homogenized material was extracted with 85% methanol containing 0.02 mM *p*-hydroxybenzyl GSL (*p*-OHB) as an internal standard. Extracts were loaded onto an anion-exchange column packed with 45 µL DEAE-Sephadex A-25^TM^ using the Millipore multiscreen column loader, and bound GSLs were desulfated overnight with sulfatase solution (Helix pomatia, Sulfatase Type H1, Sigma S9626, 0.28 mg/mL in 20 mM sodium acetate buffer). Desulfo-glucosinolates were subsequently eluted with 100 µL of MilliQ-grade water. Finally, samples were diluted 1:100 and quantified by LC-MS/MS as previously described by Jensen et al. (2015).

### Identification of GTR genes in broccoli

To identify the GTRs GTR1, GTR2, and GTR3 in *B. oleracea* L. var. *italica* (broccoli), the amino acid sequences of the corresponding *A. thaliana* transporters (AtGTR1/AtGTR2/AtGTR3), obtained from the KEGG database (https://www.genome.jp/kegg/kegg2.html), were used to perform a BLASTp search against the broccoli proteome (Braol HDEM V1.0, available at http://brassicadb.cn/#/) using the Geneious Prime platform. The resulting sequences are provided in Table S1, together with a phylogenetic analysis (Figure S2 and File S1). For tree construction, multiple sequence alignment of all GTRs was performed with Clustal Omega (EMBL-EBI). Phylogenetic reconstruction was subsequently carried out on the Phylogeny.fr platform (http://phylogeny.lirmm.fr/), employing the maximum likelihood (ML) algorithm via PhyML (v3.0). Structural protein evolution was modeled using the Whelan and Goldman substitution matrix, assuming an estimated proportion of invariant sites and a gamma distribution parameter to account for substitution rate heterogeneity among sites. The final phylogenetic tree topology was visualized and edited using iTOL (Interactive Tree Of Life v6), with branch lengths displayed to indicate evolutionary distances.

### RNA extraction

Seven-day-old broccoli seedlings, both aerial part and root from GSL-treated and control groups, were ground to a fine powder in liquid nitrogen. Total RNA was isolated using the Spectrum™ Plant Total RNA Kit (Sigma–Aldrich) following the manufacturer's protocol. RNA integrity was verified with an Agilent Bioanalyzer 2100 system (Agilent Technologies) employing the Eukaryote Total RNA Nano kit.

### RT-qPCR analysis

cDNA synthesis was carried out using 200 ng of total RNA with the *RT Master Mix for qPCR II Kit* (HY-K0510A, MedChemExpress, Sollentuna, Sweden), according to the manufacturer's instructions. Quantitative real-time PCR (RT-qPCR) was subsequently performed on an Applied Biosystems 7500 Real-Time PCR system. Each reaction consisted of 2 μL of diluted cDNA (1:100) mixed with 5 μL of 2× SYBR Green Master Mix (Applied Biosystems) and degenerate primers designed to target all broccoli GTR variants at 600 nM (primer sequences listed in Table 1), in a final reaction volume of 10 μL. For primer design, the sequences of all broccoli *GTR* paralogs identified in this study (Table S1) were aligned, and a BLAST analysis was performed to verify that the degenerate primers successfully target and bind to all variants.

**Table 1:** Primer set for gene expression analysis.

Gene name	Forward (5′-3′)	Reverse (5′-3′)
*GTR1 NPF2.10*	CTTCTATGTCGGTGGAG	CAGATCCTCAGCTAACC
*GTR2 NPF2.11*	GGGAGACCAAAGTCGTTTAC	CCAATGATCCCAAGCTTCTC
*GTR3 NPF2.9*	GATTAACCAGCTGCATCC	CCGGCGAGAAACATAATC
*18-S-RNA* ^a^	CCTGCGGCTTAATTTGACTC	AGACAAATCGCTCCACCAAC

^a^ Muries et al. 2011.

Thermal cycling conditions comprised an initial denaturation step at 95 °C for 10 min, followed by 40 cycles of 95 °C for 15 s and 60 °C for 1 min. A melt-curve analysis was included to verify amplification specificity. The same protocol was applied for both target and reference genes. Each sample was analyzed using 3 technical replicates, with 3 to 6 biological replicates per treatment. Relative transcript abundance was calculated using the 2^−ΔΔCt^ method (Livak and Schmittgen 2001).

### Library preparation and sequencing

cDNA libraries were prepared with the Illumina Stranded mRNA Prep Ligation Kit (Illumina) according to the supplier's guidelines. Library concentration was determined with the dsDNA High Sensitivity Assay on a Qubit 4 Fluorometer (Thermo Fisher Scientific), while fragment size distribution was assessed on an Agilent 2100 system using the D1000 High Sensitivity ScreenTape assay (Agilent Technologies). The resulting libraries were pooled and sequenced at the Molecular Biology Services (ACTI, University of Murcia, Spain) using the Illumina NextSeq 1000/2000 platform with 75 bp paired-end reads.

### RNA-Seq data processing and analysis

Raw Illumina RNA-Seq reads were first processed with fastp (v0.20.0) (Chen et al. 2018) to remove low-quality sequences and adapters. Read quality before and after trimming was assessed with FastQC (v0.11.9) (bioinformatics.babraham.ac.uk/projects/fastqc/). High-quality reads were aligned to the broccoli reference genome (Braol HDEM V1.0, available at http://brassicadb.cn/#/) using HISAT2 (v2.2.2) (Kim et al. 2015). Alignment files in SAM format were converted to sorted BAM files using SAMTools (v0.1.19) with default settings (Li, 2009). Gene-level counts were generated with featureCounts (v2.0.1) from the Subread package (Liao et al. 2014). For downstream analyses, normalized expression values were derived using a variance stabilizing transformation. Differentially expressed genes (DEGs) were subsequently identified with DESeq2 (Love et al. 2014), using a q-value ≤ 0.05 and log2 (fold change) thresholds of < −1 or >1.

Principal component analysis (PCA) was carried out on rlog-transformed data and visualized using the plotPCA function in DESeq2. Heatmaps were produced with the ComplexHeatmap R package (v1.20.0).

A Gene Ontology (GO) term analysis was performed to select metabolic pathways of interest. The *B. napus* multi-omics information resources (https://yanglab.hzau.edu.cn/BnIR) were used, with the var_italica_HDEM genome as a reference. Those with the most significant *P*-value and with a GeneRatio of at least 5 were selected. Broccoli gene codes (Bol) were functionally annotated based on their homology with *Arabidopsis*. These functional assignments were retrieved from TAIR and Ensembl platforms and subsequently used to categorize genes for further analytical steps.

### DNA constructs and cRNA

BolGTR1.2, BolGTR2.2, BolGTR2.3, and BolGTR3.3 (Table S1) were chosen for functional characterization based on previous RNA-Seq data generated by our group (Albaladejo-Marico et al. 2025a). The coding sequences of these selected GTRs were cloned into the pNB1 expression vector for heterologous expression in *X. laevis* oocytes, as previously described (Nour-Eldin et al. 2006). Linear transcription templates were produced by PCR ensuring amplification of the complete expression cassette including the Xenopus 5′- and 3′ UTRs. The primers used were as follows: forward primer (5´-AATTAACCCTCACTAAAGGGTTGTAATACGACTCACTATAGGG-3´) and reverse primer (5´-TTTTTTTTTTTTTTTTTTTTTTTTTTTTTATACTCAAGCTAGCCTCGAG-3´). PCR products were purified using the E.Z.N.A.® PCR Cycle Pure Kit (OMEGA Bio-Tek, Norcross, GA, USA) following the manufacturer's instructions. Purified templates were then transcribed in vitro with the mMESSAGE mMACHINE T7 Transcription Kit (Invitrogen, Thermo Fisher Scientific, Waltham, MA, USA). RNA yield was quantified using a NanoDrop spectrophotometer, and cRNAs were diluted to a final concentration of 500 to 550 ng/µL.

### *Xenopus laevis* oocytes transport assays

*X. laevis* oocytes were used to perform the transport assays following the protocol previously described by Jørgensen et al. (2017a, 2017b). Oocytes were microinjected with 50.6 nL of either cRNA or nuclease-free H_2_O using a Nanoinject II device (Drummond Scientific Company, Broomall, PA, USA). Injection needles were prepared from glass capillaries using a puller, manually trimmed with surgical scissors, and filled with mineral oil prior to use. After microinjection, oocytes were kept for 3 days at 16 °C in a pH 7.4 incubation buffer (90 mM NaCl, 1 mM KCl, 1 mM MgCl_2_, 1 mM CaCl_2_, 5 mM HEPES). After that, oocytes were preincubated for 2 min in pH 5 buffer (90 mM NaCl, 1 mM KCl, 1 mM MgCl_2_, 1 mM CaCl_2_, 5 mM MES) prior to their transfer into buffer supplemented with the 100 µM GSL mixture (3-butyl, 4-MSB, 4-MTB, 5-MSP, I3M, NMOI3M, 2-PE, p-OHB) or 100 µM GSL-B-NBD for fluorescence assays and incubated for 1 h. At the end of the incubation period, oocytes were rinsed 3 times with pH 7.4 buffer followed by a final wash with Milli-Q water. Medium samples were collected after substrate addition to oocytes. For LC-MS quantification, 1 μL of incubation medium and 1 oocyte were taken per sample, whereas for fluorescence plate reader assays, 2 μL of medium and 2 oocytes were collected. Medium fractions were handled in the same manner as oocyte extracts.

For LC-MS analysis, individual oocytes were disrupted in 62.5 μL of 50% methanol containing the internal reference compound sinigrin (1.25 μM), and extracts were stored for at least 1 h at −20 °C. Lysates were centrifuged at full speed for 10 min at 4 °C, and 50 μL of the supernatant was diluted with 75 μL Milli-Q water to yield a final methanol content of 20%, followed by filtration through 0.22 µm filter plates. Based on an estimated volume of 1 μL per oocyte, the total dilution factor was 156.25, and the internal standard concentration was 0.5 μM.

For fluorescence measurements, 2 oocytes were pooled per sample, homogenized in 110 μL Milli-Q water, and stored at −20 °C. The samples were centrifuged at maximum speed for 10 min at 4 °C, and 100 μL of the supernatant were transferred to microplates for reading. Assuming a 1 μL oocyte volume, the calculated dilution factor was 55.

### Quantification of GSLs from oocyte assays by LC-MS/MS

Oocyte samples were analyzed using a 1290 Infinity II UHPLC system coupled to an Ultivo Triple Quadrupole mass spectrometer equipped with a Jet stream ESI source operated in negative ion mode (Agilent Technologies), following a method modified from Crocoll et al. (2016). Separation was achieved on the same Kinetex XB-C18 column (100 × 2.1 mm, 1.7 µm, 100 Å, Phenomenex, Torrance, CA, USA) at 40 °C and a flow rate of 400 µL/min. The mobile phases were identical to Section 2.3.1, using the following optimized gradient: 0 to 0.2 min, 5% B; 0.2 to 3.5 min, 5% to 65% B; 3.5 to 4.2 min, 65% to 100% B; 4.2 to 4.9 min, 100% B; 4.9 to 5.0 min, 100% to 5% B; and 5.0 to 6.0 min, 5% B. MS parameters were ion spray voltage, −4,500 V; dry gas temperature, 325 °C; dry gas flow, 13 L/min; sheath gas temperature, 400 °C; sheath gas flow, 12 L/min; and nebulizer gas, 55 psi. Nitrogen was used as dry, nebulizing, and collision gas. Data processing was conducted using MassHunter Quantitation Analysis for QQQ (v10, Agilent Technologies), and detailed values for MRM transitions were monitored as previously described by (Crocoll et al. 2016).

### Confocal microscopy of fluorescent GSL uptake

To investigate the uptake of GSLs into root cells, we used a NBD tagged derivative of *p*-OHB-NBD (Figure S1e), which has been previously synthesized and characterized (Kanstrup et al. 2023a). Seven-day-old broccoli seedlings grown in vitro were rinsed 3 times with ddH_2_O, and their roots were subsequently incubated with decreasing concentrations of *p*-OHB-NBD (50, 25, 12, and 6 µM). Following incubation, roots were washed 3 additional times with ddH_2_O before staining with propidium iodide (PI, 40 µM) to visualize cell walls. Samples were then washed another 3 times to remove excess dye prior to imaging. Confocal microscopy was carried out at the Microscopy Service of the University of Copenhagen, using an SP5-X confocal laser scanning microscope. Excitation was performed at 488 nm for *p*-OHB-NBD and at 561 nm for PI. Images of roots were processed with Leica LAS X and FIJI/ImageJ.

### Statistical analysis

Statistical analyses and data presentation were conducted using Origin (Pro), Version 2021 (OriginLab Corporation, Northampton, MA, USA). Tests were preceded by a normality test and a Grubbs test to identify potential outliers. All the data were subjected to a *t*-test. Significance levels are denoted as follows: * for *P* < 0.05, ** for *P* < 0.01, *** for *P* < 0.001, and n.s. for no significant differences. All presented values represent the means ± SE. Heatmaps were generated using R 4.3.1 software (R Core Team 2021). Specifically, gplots (Warnes et al. 2016) and pheatmap packages (Kolde 2012) were used.

## Results

### In vitro growth stimulation by exogenous GSLs

The application of exogenous GSLs, specifically 4-MSB (0.08 µM) and I3M (2.53 µM), in the agar medium significantly increased both FW and DW of broccoli seedlings compared with the untreated control after 7 days of exposure (Fig. 1).

**Figure 1:**
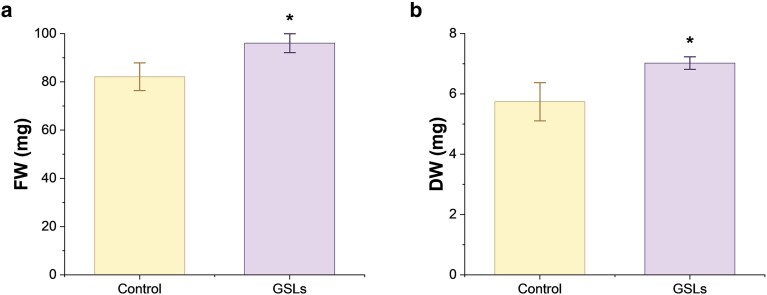
Effect of exogenous GSLs on broccoli seedling growth in vitro. (a) Fresh weight (FW) and (b) dry weight (DW) of broccoli seedlings after 7 days of growth. The yellow bars represent untreated control plants, while the purple bars correspond to seedlings treated with pure GSLs (4-MSB, 0.08 µM; I3M, 2.53 µM). Each bar represents the mean ± SE (n = 20 for FW and n = 10 for DW). Significant differences between treated seedlings and the control were determined via *t*-tests. * *P* < 0.05, ** *P* < 0.01, *** *P* < 0.001.

### Decrease of GSLs in broccoli seedlings

Glucosinolate levels were quantified in whole broccoli seedlings at Days 1 and 3 and separately in the aerial part and roots at Days 5 and 7 after in vitro germination on agar (Fig. 2). Overall, a general decrease in the endogenous content of the different GSLs measured was observed over time in seedlings treated with GSLs compared to the control. On Day 1, the content of the aliphatic GSLs 3-methylthiopropyl (3-MTP), 3-methylsulfinylpropyl (3-MSP), and 5-methylthiopentyl decreased, together with the aromatic GSL benzyl. By Day 3, a reduction was also observed in the aliphatic GSLs 4-methylthiobutyl (4-MTB) and 4-MSB, as well as in the indolic GSL N-methoxyindol-3-ylmethyl (NMOI3M). At later stages (Days 5 and 7), changes in GSL levels were more pronounced in roots than in the aerial part. Specifically, on Day 5, root levels of 3-MTP, 3-MSP, 4-MSB, 5-methylsulfinylpentyl (5-MSP), I3M, and NMOI3M decreased, whereas an increase in 6-methylsulfinylhexyl was detected in the aerial part. Finally, on Day 7, all quantified GSLs decreased in the roots, while further reductions of 3-MTP, 4-MTB, and benzyl were also observed in the aerial part.

**Figure 2:**
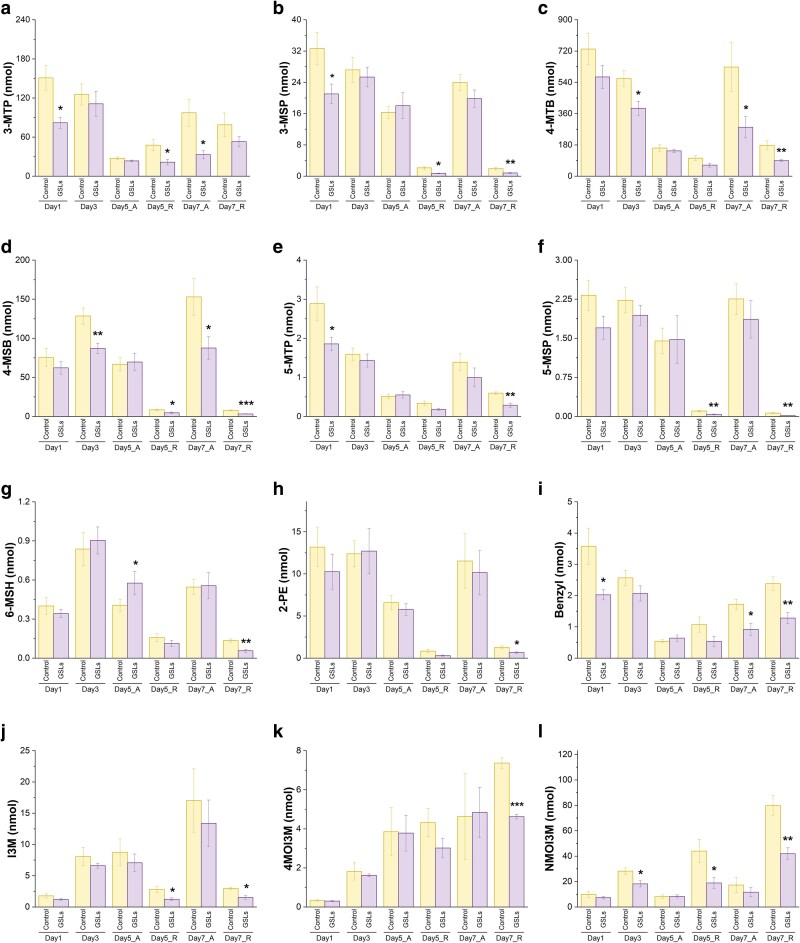
Glucosinolate profiles in broccoli-treated seedlings at Days 1 and 3 and in the aerial part and roots at Days 5 and 7. Content of aliphatic (a) 3-methylthiopropyl (3-MTP), (b) 3-methylsulfinylpropyl (3-MSP), (c) 4-methylthiobutyl (4-MTB), (d) 4-methylsulfinylbutyl (4-MSB), (e) 5-methylthiopentyl (5-MTP), (f) 5-methylsulfinylpentyl (5-MSP), (g) 6-methylsulfinylhexyl (6-MSH), aromatic (h) 2-phenylethyl (2-PE), (i) benzyl glucosinolate, and indolic (j) indol-3-ylmethyl (I3M), (k) 4-methoxyindol-3-ylmethyl, (l) N-methoxyindol-3-ylmethyl (NMOI3M). Values represent the endogenous GSL content (nmol) extracted from a standardized and identical fresh tissue weight across all samples. The yellow bars represent untreated control plants, while the purple bars correspond to seedlings treated with purified GSL (4-MSB, 0.08 µM; I3M, 2.53 µM). Each bar represents the mean ± SE (n = 6). Significant differences between treated seedlings and the control were determined via *t*-tests. * *P* < 0.05, ** *P* < 0.01, *** *P* < 0.001.

### Upregulation of GTR transporter following GSL treatment

The transcriptional response of the main GTRs, *GTR1*, *GTR2*, and *GTR3*, was evaluated by qPCR in roots of broccoli seedlings treated with GSLs compared with untreated controls (Fig. 3). GSL-treated seedlings exhibited higher transcript levels of *GTR2* and *GTR3* at Day 3 and a sustained upregulation of *GTR3* at Day 5. By Day 7, no significant differences in expression were detected for any of the analyzed transporters.

**Figure 3:**
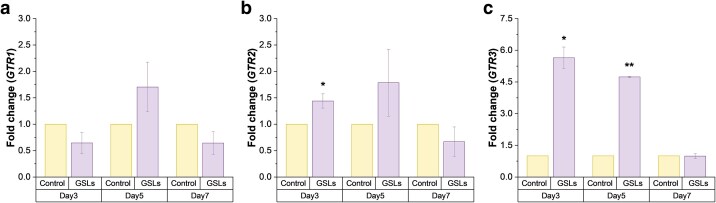
Transcript levels of glucosinolate transporters (GTRs) in control and treated roots of broccoli seedlings at Days 3, 5, and 7. Expression of (a) GTR1, (b) GTR2, and (c) GTR3. The yellow bars represent untreated control plants, while the purple bars correspond to seedlings treated with purified GSLs (4-MSB, 0.08 µM; I3M, 2.53 µM). Each bar represents the mean ± SE (n = 3 to 6). Significant differences between treated seedlings and the control were determined via *t*-tests. * *P* < 0.05, ** *P* < 0.01, *** *P* < 0.001.

### GSL transport activity of GTRs heterologously expressed in *Xenopus* oocytes

Four GTRs (GTR1.2, GTR2.2, GTR2.3, and GTR3.3) were selected to evaluate their capacity to transport GSLs in *X. laevis* oocytes (Fig. 4). Oocytes expressing each transporter were exposed to a 100 µM equimolar mixture of GSLs at pH 5 for 1 h after which uptake was determined by LCMS. All tested transporters were able to actively transport GSLs, as evidenced by their capacity to accumulate these compounds against a concentration gradient. Overall, GTR1.2 and GTR2.2 displayed the highest transport efficiency for all classes of GSLs (aliphatic, indolic, and aromatic), followed by GTR2.3.

**Figure 4:**
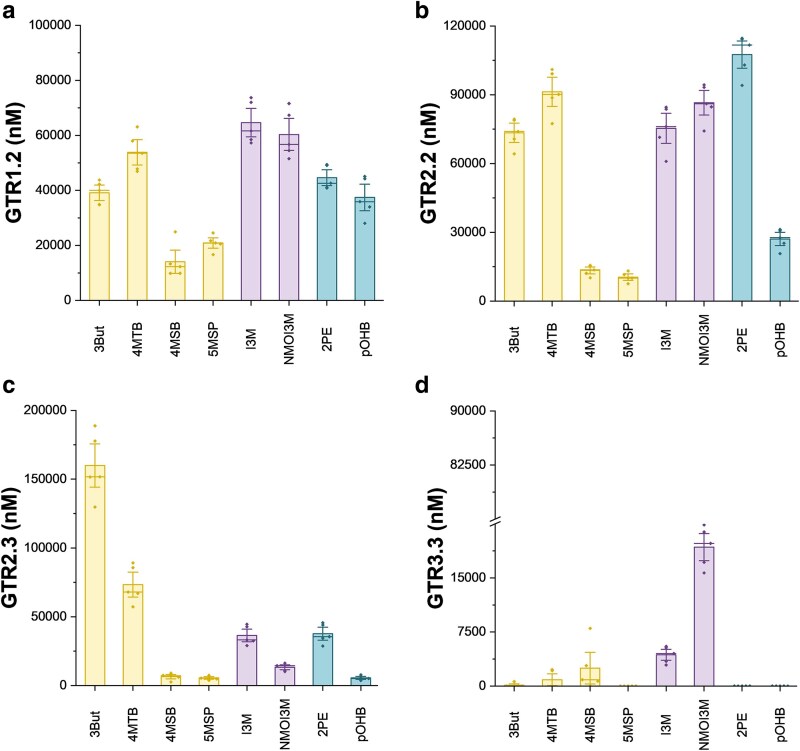
Functional characterization of broccoli GTRs in a heterologous system. LCMS analysis of GSL uptake by (a) GTR1.2, (b) GTR2.2, (c) GTR2.3, and (d) GTR3.3 expressed in *Xenopus laevis* oocytes. The yellow bars indicate aliphatic GSLs, purple bars indicate indolic GSLs, and blue bars indicate aromatic GSLs. Each bar represents the mean ± SE (n = 5).

GTR1.2 showed the highest uptake levels for 4-MTB, I3M, and NMOI3M, whereas GTR2.2 preferentially transported 3-But, 4-MTB, and NMOI3M, and GTR2.3 showed higher transport of 3-But, 4-MTB, and I3M. In contrast, GTR3.3 exhibited a clear preference for indolic GSLs, such as I3M and NMOI3M. In Figure S3, the relative transport activity of each transporter toward the different compounds is shown, in comparison with mock-injected oocytes (negative control) and AtGTR1-expressing oocytes (positive control).

### Internalization of fluorescent GSLs by broccoli root

The ability of 7-day-old broccoli seedlings to internalize the fluorescent GSL (F-GSL) *p*-OHB-NBD was examined using seedlings grown in vitro on agar, in which the F-GSL *p*-OHB-NBD was supplied exclusively via the root system. When the roots of broccoli seedlings were exposed to a 50 µM solution of *p*-OHB-NBD, fluorescent deposits were clearly observed inside root cells (white arrows in Fig. 5b). This phenomenon was also detected, although with lower intensity, when roots were incubated with a 25 µM solution (Fig. 5e). As expected, fluorescence intensity progressively decreased as the concentration of F-GSL was reduced (Fig. 5k). To assess cellular location, roots of intact seedlings were also stained with PI to label viable cells and subsequently incubated with the F-GSL (Fig. 5h, i). The merged image (Fig. 5j) shows the overlap between PI and F-GSL signals, indicating that the F-GSL accumulates within root cells rather than remaining associated with the root surface.

**Figure 5:**
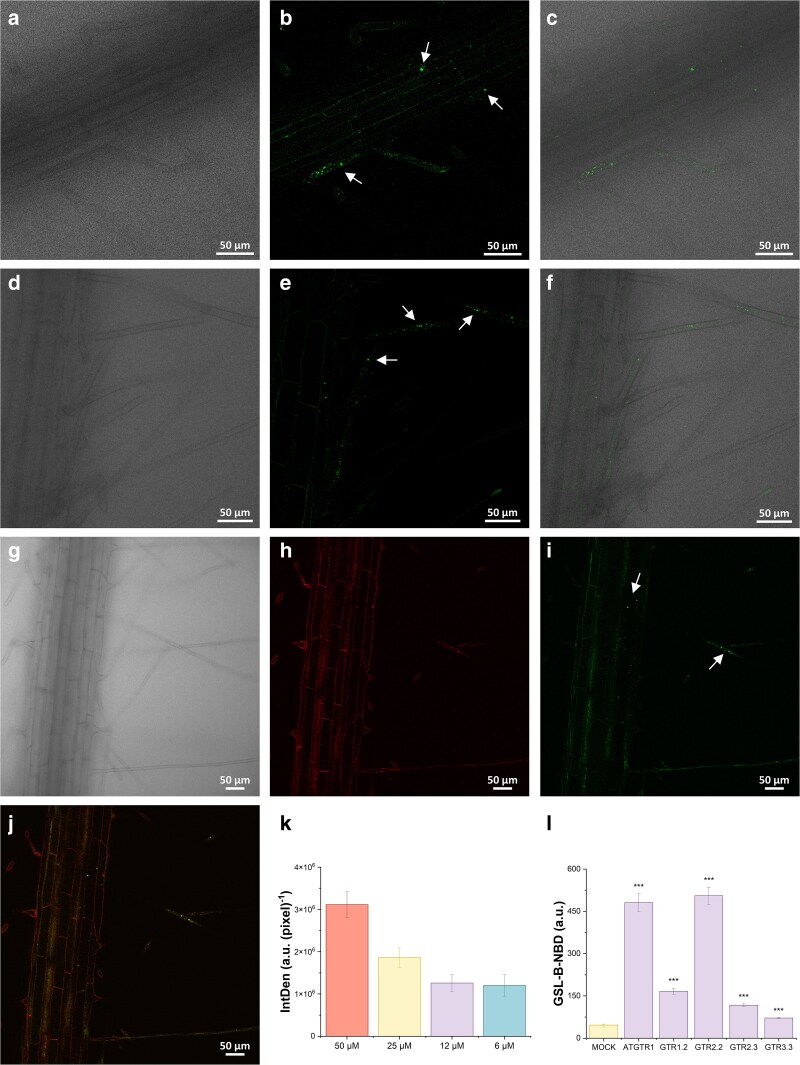
Application of the fluorescent glucosinolate (F-GSL) p-OHB-NBD to 7-day-old broccoli seedlings observed by confocal microscopy. (a–c) Root of broccoli seedlings treated with 50 µM F-GSL: transmitted light/root image (a), F-GSL fluorescence channel (b), and merged image (c). (d–f) Root of broccoli seedlings treated with 25 µM F-GSL: transmitted light/root image (d), F-GSL fluorescence channel (e), and merged image (f). (g–j) Root of broccoli seedlings treated with 50 µM F-GSL and 40 µM propidium iodide (PI): transmitted light/root image (g), PI fluorescence channel (h), F-GSL fluorescence channel (i), and merged image (j). (k) Quantification of F-GSL fluorescence intensity. Each bar represents the mean ± SE of 6 biological replicates and 2 technical replicates. (l) Plate-based analysis of fluorescent glucosinolate (F-GSL) p-OHB-NBD uptake by AtGTR1, GTR1.2, GTR2.2, GTR2.3, and GTR3.3 and water-injected MOCK oocytes expressed in *Xenopus laevis*. Each bar represents the mean ± SE (n = 5). Significant differences between GTR-expressing oocytes and the MOCK control were determined by *t*-tests (* *P* < 0.05, ** *P* < 0.01, *** *P* < 0.001).

Negative controls are shown in Figure S4. Roots were incubated with the fluorophore alone (NBD) at 50 µM (Figure S4b) and 25 µM (Figure S4d) to confirm that internalization occurred only when the fluorophore was conjugated to the GSL and that free NBD was not passively taken up by the cells. An additional negative control, in which root cells were stained only with PI (Figure S4h), showed no fluorescence in the F-GSL channel (Figure S4i), confirming the specificity of the signal.

Finally, the ability of the broccoli GTRs analyzed in this study (GTR1.2, GTR2.2, GTR2.3, and GTR3.3) to transport the F-GSL was evaluated in a *X. laevis* oocyte heterologous system (Fig. 5l). Oocytes expressing GTR2.2 showed the highest accumulation of F-GSL, reaching levels comparable to those observed for AtGTR1, which was included as a positive control (previously validated by Kanstrup et al. (2023a). GTR1.2-, GTR2.3-, and GTR3.3-expressing oocytes displayed intermediate fluorescence signals that were significantly higher than those detected in water-injected oocytes (MOCK), used as a negative control.

### Identification of GSL-responsive genes

Transcriptomic analysis was performed on 7-day-old broccoli seedlings treated with GSLs under in vitro conditions (Figure S5). A total of 61,279 genes were analyzed. Using an adjusted *P*-value < 0.05 as the statistical threshold, 807 genes were identified as significantly affected by GSL treatment, including 776 genes in roots and 31 genes in aerial tissues. After applying an additional effect-size threshold of |fold change| > 1, 54 genes were retained for downstream interpretation, comprising 38 genes in roots and 16 genes in aerial tissues. In the aerial part, this corresponded to 2 upregulated and 14 downregulated genes in response to GSL treatment (Figure S5b). In contrast, the roots showed a stronger transcriptional response, with 27 upregulated and 11 downregulated genes (Figure S5d). A complete list of these DEGs, including their respective Bol identifiers and available functional annotations, is provided in Table S2. Principal component analysis of both tissues (Figure S5a and c) clearly separated control and GSL-treated samples into 2 distinct clusters, indicating consistent transcriptional differences between treatments.

Genes with an *adjusted P-value < 0.05* were further examined to explore the transcriptional response associated with GSL treatment (Fig. 6). In the aerial part, the heatmap showed a limited transcriptional response, mainly characterized by the downregulation of a small set of genes in GSL-treated seedlings compared with controls (Fig. 6a). Consistently, GO enrichment analysis revealed that these downregulated genes were primarily associated with aging-related processes (GO:0007568) (Fig. 6c). In the roots, the heatmap revealed a clearer separation between control and GSL-treated seedlings, with a subset of genes showing marked upregulation in response to GSL treatment (Fig. 6b). Gene Ontology enrichment analysis (Fig. 6d) revealed that several biosynthetic and metabolic pathways were upregulated, including secondary metabolism (GO:0044550), GSL metabolism (GO:0019761), plant-type cell wall organization (GO:0009505), Golgi-associated processes (GO:0000138, GO:0005795), and secretory vesicle functions (GO:0099503). Conversely, fatty acid metabolism (GO:0006631), phenylpropanoid pathways (GO:0009699), as well as processes linked to cell death (GO:0008219) and aging (GO:0007568) were downregulated. This structural reprogramming was characterized by an upregulation of genes associated with cell wall glycoprotein adhesion (*AGP31*), wall loosening and elongation (*XTH*), synthesis and remodeling of polysaccharides (*PAE8*, *GAE*), and intracellular signaling and vesicular trafficking (*ROPGAP4*, *REN1*, *PRA1.B2*).

**Figure 6:**
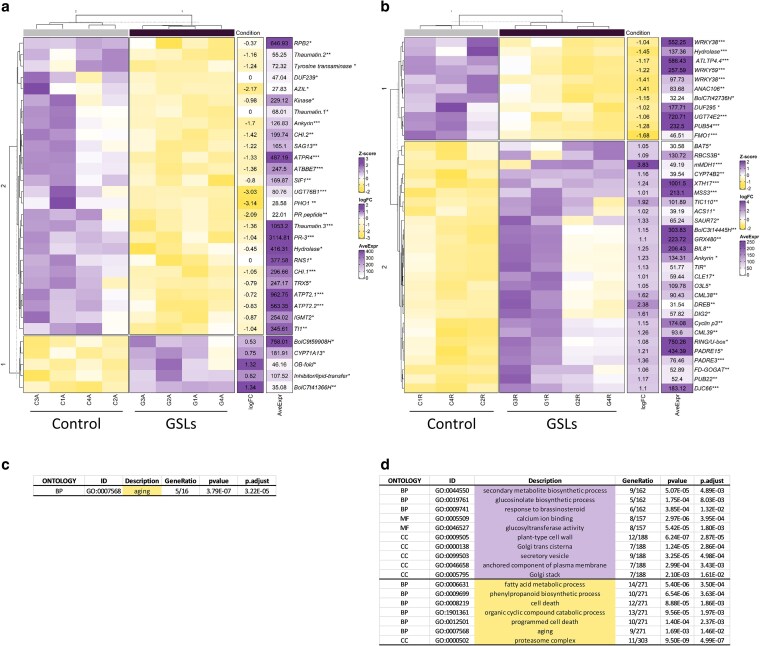
Functional analysis of GSL-responsive genes. Heatmaps showing GSL-responsive genes in aerial parts (a) and roots (b), together with their associated Gene Ontology (GO) enrichment results (c, d). For aerial tissues, the heatmap includes genes with *P* < 0.05, whereas for roots, an additional effect-size threshold of |logFC| > 1 was applied to highlight the strongest transcriptional changes. GO enrichment analysis was performed using genes with *P* < 0.05 in both tissues. Yellow indicates genes with low expression and purple indicates genes with high expression (Z-score). Fold change (logFC), mean expression (AveExpr), and gene names are shown. Significant differences between GSL-treated and control plants were determined using *t*-tests: *P < 0.05, **P < 0.01, ***P < 0.001.

### Upregulation of GSL-related genes in response to GSL treatment

Genes involved in GSL biosynthesis and regulation were significantly affected in the roots of 7-day-old broccoli seedlings treated with exogenous GSLs compared to untreated controls (Fig. 7). The aliphatic GSL biosynthetic pathway was the most strongly affected, showing upregulation of key genes including *BCAT4*, *BAT5*, *CYP79F1*, *CYP83A1*, and *UGT74C1*, which are associated with core structure formation and side-chain elongation. In the indolic GSL pathway, the transcription factor *MYB34* and the biosynthetic enzyme *CYP79B2* were also upregulated. In contrast, *APR2*, a gene involved in sulfur assimilation and reduction, was downregulated in response to GSL treatment.

**Figure 7:**
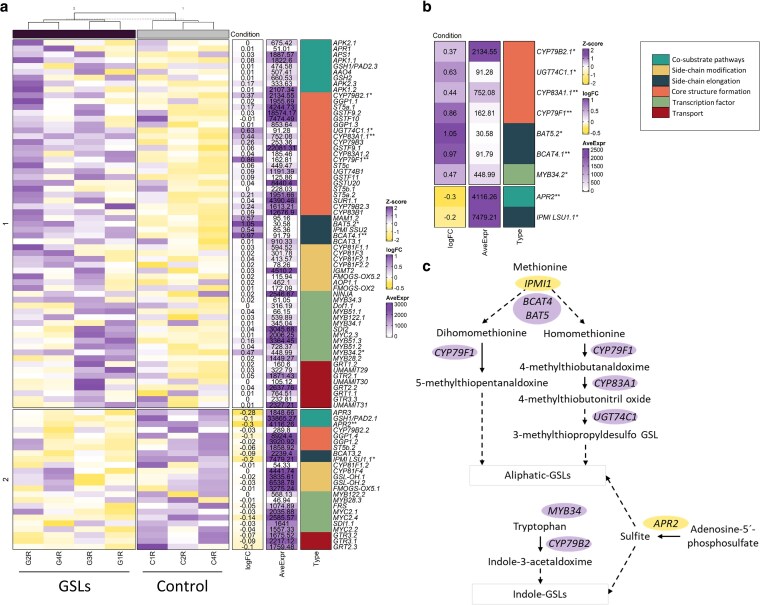
GSL biosynthesis and regulatory genes in roots of broccoli seedlings treated with exogenous GSLs. (a) Heatmap showing the expression of all analyzed genes associated with GSL metabolism in broccoli seedling roots. (b) Heatmap of differentially expressed GSL-related genes with *P*-value < 0.05. Yellow indicates genes with low expression and purple indicates genes with high expression (Z-score). Fold change (logFC), mean expression (AveExpr), and gene names are shown. Statistical differences between GSL-treated and control plants were determined using *t*-tests (*P < 0.05, **P < 0.01, ***P < 0.001). (c) Schematic representation of the GSLs biosynthetic pathway highlighting affected genes; downregulated genes are shown in yellow and upregulated genes in purple. Solid arrows indicate direct biochemical conversions between compounds, whereas dashed arrows denote pathway steps involving intermediate reactions that are not explicitly shown in the diagram.

## Discussion

To address the knowledge gap arising from our previous results, which demonstrated that the exogenous application of GSLs enhances crop growth and abiotic stress resistance (Albaladejo-Marico et al. 2024, 2025b, 2026), this study investigates how exogenously supplied GSLs (4-MSB and I3M) are taken up by intact broccoli seedlings via the root system. These results are associated with measurable physiological and transcriptional responses during early development (7 days). Furthermore, by combining heterologous expression in *Xenopus laevis* oocytes, GSL quantification, fluorescence microscopy, and RNA-sequencing techniques, we provide evidence for the active transport and metabolic integration of these compounds.

The exogenous application of GSLs significantly promoted seedling development, as evidenced by the increase in both FW and DW (Fig. 1). This phenotype was supported at the transcriptomic level by the enrichment of GO categories related to cell wall organization, Golgi-associated processes, secretory vesicle function, secondary metabolism, and GSL metabolism (Fig. 6), suggesting that GSL internalization is associated with root structural reprogramming and metabolic adjustment during seedling establishment (Toyooka et al. 2009; Huot et al. 2014). The downregulation of fatty acid metabolism and phenylpropanoid biosynthesis suggests a shift in metabolic priorities that may contribute to the growth-promoting phenotype. Upon application of these external GSLs, a decrease in endogenous GSL levels was observed (Fig. 2). This overall reduction during the first days after germination suggested that GSLs were actively consumed and/or remobilized during early seedling establishment. In *Brassica* seeds and seedlings, GSLs have been reported to function not only as defense metabolites but also as reservoirs of sulfur (Soudthedlath et al. 2025; Ali et al. 2025). Their early depletion is consistent with the high metabolic demands associated with rapid growth and tissue differentiation during seedling establishment (Bellostas et al. 2007; Ali et al. 2025). Regarding these endogenous profiles, both control and treated seedlings showed the expected developmental trend, characterized by a progressive decrease in aliphatic GSLs and a concomitant increase in indolic GSLs. This architectural diversity has been found closely linked to distinct physiological roles (Malhotra et al. 2023): sulfur-rich aliphatic GSLs may act as stable nutrient reservoirs (Zhang et al. 2020), while indolic GSLs are tightly associated with defense signaling and cell expansion cross talk (Clay et al. 2009). Interestingly, the endogenous GSL profile did not mirror the composition of the external medium. Although I3M was supplied at a substantially higher concentration than 4-MSB (30:1 ratio), aliphatic GSLs remained the predominant class within the seedlings. This observation further supports the finding that exogenous GSLs do not simply accumulate in proportion to their external availability but rather are integrated into a tightly regulated endogenous metabolic network. Moreover, the endogenous reduction of GSLs was more pronounced in seedlings treated with exogenous GSLs than in untreated controls. This depletion occurred concomitantly with the progressive disappearance of GSLs from the growth medium between Days 3 and 7, as previously reported in our agar-based system (Albaladejo-Marico et al. 2025a, 2025b). This result again supports the hypothesis that external GSL uptake and/or perception may trigger an active turnover and metabolic reorganization of the endogenous GSL pool. Together with the transient induction of GTR transporters detected at early stages (Fig. 3), these observations are consistent with a dynamic GSL exchange and homeostatic adjustment during seedling growth. This metabolic redirection was further supported by our GO enrichment analysis in the root, which showed a significant upregulation of genes related to biosynthetic and metabolic pathways in GSL-treated seedlings compared with controls (Fig. 6b).

Germination and early seedling establishment have been described as a critical high-demand stage, where the rapid transition from heterotrophic to autotrophic growth requires the immediate mobilization of nutrient reserves (Ha et al. 2017). Our results supported a model where GSLs act as a strategic sulfur buffer during this period. As proposed by Sugiyama et al. (2021), 4-MTB functions as a stable storage reservoir, while 3-MTP serves as a more accessible metabolic pool that is prioritized for degradation to supply the cysteine biosynthesis pathway. In broccoli seedlings, the early depletion of 3-MTP on Day 1 (Fig. 2a) aligns with this intensive metabolic demand, providing the necessary S-containing groups to support root elongation and protein synthesis before primary S-assimilation (via *APR2*) is fully required or optimized. This is particularly relevant as APR2 has been reported as the major isoform contributing to total APR activity in Brassicaceae (Yatusevich et al. 2010).

Although seedlings on agar rely primarily on seed reserves, the assimilation of inorganic sulfate, whether derived from inevitable catabolic oxidation of internal sulfur pools or trace external sources, remains an energetically expensive process, requiring significant amounts of ATP and reducing power (NADPH) to convert inorganic sulfate into organic compounds (Takahashi et al. 2011; Kopriva et al. 2012). The downregulation of *APR2* in GSL-treated plants (Fig. 7) suggests a strategic energy-saving mechanism. By preferentially remobilizing GSLs, which already contain sulfur in a reduced form, the seedling bypasses the costly *APR2*-mediated pathway. This energetic “shortcut” is particularly advantageous during the high-demand stage of germination, allowing the plant to reallocate its limited energy reserves toward rapid cell wall remodeling (Figure S6) and biomass accumulation (Fig. 1).

To facilitate this strategic mobilization and the uptake of exogenous GSLs, the seedling modulates its transport capacity. This process is likely to be genotype-dependent, as *Brassica* species display extensive diversity in GSL profiles (Windsor et al. 2005), determined by both biosynthetic gene repertoires and transporter specificity (Kliebenstein and Cacho 2016; Mann et al. 2023; Ramos et al. 2025). Therefore, the physiological outcome of exogenous GSL application may depend not only on the amount of GSLs supplied but also on the specific GSL molecular species present and the transporters available to internalize and redistribute them. In fact, recent structural and functional studies have revealed that GTRs are not passive carriers but highly selective transporters whose substrate recognition properties directly influence GSL allocation throughout the plant (Kanstrup et al. 2023b; Nour-Eldin et al. 2025). Although all GTRs recognize the conserved GSL core, differences in substrate affinity and transport efficiency among GSL classes provide a mechanistic basis for the distinct behavior of individual GSL species observed in this study.

In this context, our analysis showed a significant upregulation of *GTR2* and *GTR3* at Days 3 and 5 in GSL-treated roots (Fig. 3b, c). This temporal window is critical, as it coincides with the period when the major “storage pool” (4-MTB) begins to be depleted and may reflect the demand for sulfur and signaling molecules peaks. The induction of *GTR2*, a high-affinity transporter for aliphatic GSLs, and *GTR3*, which shows preference for indolic GSLs, suggests a coordinated effort to maximize the internal pool of both GSL classes. This is further supported by the high transport efficiency of GTR2.2 for aliphatic GSLs and the clear preference of GTR3.3 for indolic compounds (such as I3M and NMOI3M), which mirror the substrate specificities previously described for their orthologs in *A. thaliana* (Jørgensen et al. 2017a, 2017b). This evolutionary conservation of GTR function underscores their role in GSL homeostasis. Interestingly, by Day 7, the expression of these transporters returned to control levels, showing no significant differences. This transient transcriptional response suggests that *GTR* induction is an early and reversible response to GSL treatment, likely associated with the reestablishment of GSL homeostasis during seedling development. Nevertheless, it is important to highlight that different broccoli GTR paralogs (Figure S2) display distinct substrate affinities and specificities toward various GSLs (Fig. 4), suggesting they may exhibit unique sub-functionalization, tissue-specific expression, or kinetic properties (Kanstrup et al. 2023b; Nour-Eldin et al. 2025). Therefore, while our data reflect a coordinated overall transport response, further subfamily-specific resolution is necessary to fully dissect the precise individual contribution of each broccoli paralog to early seedling development. As it has been reported that transcript abundance does not necessarily correlate with functional protein levels or activity (Velez-Bermudez and Schmidt 2014), future studies focusing on GTR protein dynamics and posttranslational regulation will also be essential to determine whether transporter activity persists after the transcriptional signal declines. From a growthpromotion perspective, these results suggest that the effect of exogenous GSLs may depend on the balance between aliphatic (nutrient reservoirs) and indolic compounds (defense signaling and cell expansion crosstalk) (Malhotra et al. 2023). Thus, the relative composition of GSL-based treatments should be considered according to the intended physiological outcome and environmental context, such as growth promotion under nonstress conditions or improved tolerance under abiotic stress (Albaladejo-Marico et al. 2024, 2026). In this sense, our results showed that the chemical specificity of GTR-mediated transport provided a mechanistic basis to explain why different GSL molecular species may have distinct physiological effects during seedling establishment.

The relevance of the transcriptional upregulation of GTRs was further supported by our functional characterization in the *Xenopus laevis* heterologous system. Our results in oocytes (Fig. 4) confirmed that broccoli GTR1.2, GTR2.2, GTR2.3, and GTR3.3 are fully functional transporters capable of active GSL uptake against a concentration gradient, consistent with their established roles in GSL translocation in *Brassicaceae* species (Andersen and Halkier 2014; Chung et al. 2022). While in *Arabidopsis* these transporters have been primarily linked to long-distance translocation and source-sink distribution (Nour-Eldin et al. 2012), our data in broccoli suggest an additional, vital role during the seedling stage: facilitating the active entry of exogenous GSLs from the rhizosphere to satisfy the immediate metabolic and signaling requirements of the developing roots. Our results in broccoli align with the model proposed by (Andersen et al. 2013), where GTR1 and GTR2 mediate the bidirectional transport of aliphatic GSLs, confirming that the root is an organ with a robust active import capacity. In our system, this mechanism allows the seedling to “capture” external resources to promote its intensive growth phase, bypassing the energetic costs of primary assimilation.

To unequivocally link GTR activity with cellular uptake, we utilized a fluorescent glucosinolate derivative (F-GSL, *p*-OHB-NBD) (Kanstrup et al. 2023a). The successful translocation of this compound in the *Xenopus* system (Fig. 5l) provides biochemical validation that the specific broccoli GTRs identified in our study retain substrate recognition capabilities even when the GSL is modified. Although the overall transport patterns were consistent with those previously observed for the native *p*-OHB GSL (Figure S3h), minor differences in transporter preferences were detected. These discrepancies likely arise from differences between the experimental setups. Previous characterization assays were performed using a competitive mixture of native aliphatic, indolic, and benzylic GSLs, whereas the fluorescent derivative was supplied as a single substrate. Under competitive conditions, transport reflects the relative affinity of each GTR for a given GSL in a mixed environment, while single-substrate assays reveal the maximum intrinsic transport capacity of individual transporters in isolation for a specific metabolite. In addition, the presence of the NBD fluorophore may slightly alter substrate–transporter interactions, contributing to the observed differences in transport efficiency. Translating this to an in vivo context, our confocal microscopy data provide direct spatial evidence of active internalization. The intracellular accumulation of F-GSL in viable root cells (Fig. 5j), contrasted with the complete exclusion of the free fluorophore (NBD) (Figure S4), rules out passive diffusion mechanisms. Instead, this specificity underscores that uptake is strictly dependent on the recognition of the intact GSL moiety, further supporting the role of GTRs as the primary gateways for exogenous GSL entry.

Nevertheless, following this initial consumption, our transcriptomic data at Day 7 revealed a secondary phase in this metabolic strategy: a robust upregulation of the core GSL biosynthetic machinery (such as *BCAT4*, *BAT5*, *CYP79F1*, *MYB34*, and *CYP79B2*) (Fig. 7). This delayed induction suggested a compensatory feedback mechanism. Once the initial “metabolic pool” (3-MTP) and even the “storage pool” (4-MTB) have been depleted to support early growth, the seedling must transition from consumption to active production to reestablish GSL homeostasis and ensure future chemical defense. These results align remarkably with previous findings from our group, where broccoli seeds primed with a GSL-rich extract exhibited a parallel molecular signature: a downregulation of *APR2* concomitant with the upregulation of biosynthetic genes. Notably, that study also reported a consistent decrease in endogenous 4-MSB and I3M levels across early developmental stages (Days 1, 3, 7, and 10) (Albaladejo-Marico et al. 2025a).

Collectively, these findings reinforce the hypothesis that GSLs are not merely defensive compounds but also dynamic metabolic reserves, whose strategic remobilization allows for the reallocation of cellular energy toward active growth. This is consistent with the model proposed by Nour-Eldin et al. (2012), who demonstrated that *gtr1 gtr2* double mutants are unable to effectively mobilize GSL-derived sulfur, leading to severe growth defects under sulfur-deficient conditions. Such evidence confirms that while seedlings ultimately depend on exogenous sulfur for sustained growth, GSLs serve as a critical internal buffer that supports the plant during the high-demand transition of early establishment. However, it should be noted that this transcriptomic analysis was performed exclusively at Day 7, representing a relatively late stage of seedling establishment. Given that significant changes in endogenous GSL levels and *GTR* transporter expression were already detected during the first days after treatment, future studies focusing on earlier transcriptional responses will be essential to better resolve the initial signaling and metabolic events triggered by exogenous GSLs.

Beyond metabolic adjustments, our transcriptomic analysis revealed that exogenous GSLs trigger a strong structural reprogramming in the root. We detected an induction of genes associated with cell wall remodeling and matrix restructuring. The upregulation of *XTH4*, a marker for wall loosening (Kushwah et al. 2020), along with *PAE8* and *GAE6*, involved in pectin and polysaccharide modification, points toward an increase in cell wall plasticity (Souza et al. 2014; Bethke et al. 2016). Furthermore, the induction of arabinogalactan proteins such as *AGP31* suggests enhanced communication between the wall and the plasma membrane (Hijazi et al. 2014). Together, these results revealed that GSLs did not merely accumulate passively, as it has been recently reported as acting as signaling molecules capable of modulating intercellular trafficking (Fernandez et al. 2025). In our broccoli seedlings, this signaling role appears to activate the cellular machinery for expansion. The structural softening may be involved in the significant increase in biomass observed in our study, as a more plastic root system can more efficiently access resources in the growth medium (Kumar et al. 2019; Amrani 2023).

In conclusion, we demonstrate that broccoli seedlings actively uptake exogenous GSLs via GTR transporters, effectively crossing mature apoplastic barriers to trigger a strong biostimulant response. Our findings revealed that GSLs function as more than just sulfur reservoirs; they act as metabolic rheostats that coordinate an energetic “shortcut” by bypassing the costly primary sulfur assimilation pathway. This reallocation of energy and sulfur resources drives a biphasic developmental program: an initial phase of intensive GSL mobilization to support rapid growth, followed by the transcriptional activation of de novo biosynthesis to reestablish chemical defense homeostasis. Furthermore, the induction of cell wall remodeling genes suggests that GSLs serve as signaling molecules that modulate root structural plasticity. This integrated response ensures the efficient mobilization and redistribution of sulfur resources to support the critical stages of early development, ultimately enhancing seedling establishment and biomass accumulation.

## Supplementary Material

kiag556_Supplementary_Data

## Data Availability

The omics data that support the findings of this study are openly available in NCBI at reference number PRJNA1446223. The other data that support the findings of this study are available from the corresponding author upon reasonable request. The author responsible for distribution of materials integral to the findings presented in this article in accordance with the policy described in the Instructions for Authors is L.Y.M.
