# Supplementary material for: Beyond defense: GTR-mediated uptake of glucosinolates drives growth and structural reprogramming in broccoli seedlings
Source: Plant Physiol. 2026 Jul 30;201(4):kiag556. doi: 10.1093/plphys/kiag556 (PMC13464505; doi:10.1093/plphys/kiag556)
Supplement: Supplementary file 1 [file kiag556_supplementary_data.zip › supplementary file 1.pdf]

CLUSTAL O(1.2.4) multiple sequence alignment

```
Bol_GTR33      ---------------------------------------------------MEAEKTE     7
AT_GTR3        ---------------------------------------------------MEVEKTE     7
Bol_GTR31      ---------------------------------------------------MEVE-KN     6
Bol_GTR32      ---RILF------------KLFLQSYLTI---KLKCF----------------IPSQ-EN   25
Bol_GTR23      -------------------------------MERKSLEVESM-NHQKLSSAVYDGSVTAV   28
AT_GTR2        -------------------------------MERKPLELESTDNHQNPSSAVYGGSVTAV   29
Bol_GTR21      -------------------------------MERNPLEVESTD-HQKPSSAVYDGSATAV   28
Bol_GTR22      -------------------------------MERKPLEVESTD-HQKPPSAVYDGSVMAV   28
AT_GTR1        MKSRVILNHRDRRDKNHNNN-NTNHYTQVDTMERKPLEVEPSTTT---------TNTDV-   49
Bol_GTR11      MKSRVILSHRERRDK--------HNYTQIETMERKPFDVETTEDHKAYSSVDGGG-SDS-   50
Bol_GTR12      -----FLSHRERRDKNNNQDISCNNYTQIETMERKPFEVETTEDHKPYSDGGGSDLTST-   54


Bol_GTR33      KKITEEEDDESKIIYRGWKVMPFIIGNETFEKLGIVGSSSNLVIYLTTVFNMKSITAATV   67
AT_GTR3        --KNIAEDDESKIIYRGWKVMPFIIGNETFEKLGIVGSSSNLVIYLTTVFNMKSITAAKV   65
Bol_GTR31      --ITGEDDDESKLIYRGWKVMPFIIGNETFEKLGIVGSSSNLVIYLTTVFNMKSITAATV   64
Bol_GTR32      --TTGGEDDESKIIYRGWKVMPFIIGNETFEKLGIVGSSSNLVIYLTTVFNMKSITAATV   83
Bol_GTR23      DSVKEDVQE-TKVVYRGWKVMPFIIGNETFEKLGIIGTLSNLLVYLTSVFNMKSVTAATI   87
AT_GTR2        DSVEEDVQNQKKVVYRGWKVMPFIIGNETFEKLGIIGTLSNLLVYLTAVFNLKSITAATI   89
Bol_GTR21      DSVEEEVQE-TKLVYRGWKVMPFIIGNETFEKLGIIGTLSNLLVYLTAVFNMKSITAATI   87
Bol_GTR22      DSVEEEVGE-TKVVYRGWKVMPFIIGNETFEKLGIIGTLSNLLVYLTAVFNMKSITAATI   87
AT_GTR1        --VDSFEEEQRKIVYRGWKVMPFIIGNETFEKLGIIGTLSNLLVYLTSVFNLKSYTAATI   107
Bol_GTR11      --VDSFEDEQKKLVYRGWKVMPFIIGNETFEKIGIIGTLSNLLVYLTSVFNLQSVTAATI   108
Bol_GTR12      --VDSSGDEQKKLVYRGWKVMPYIIGNETFEKIGIIGTLSNLLVYLTQVFNLKKYTAATI   112
                       :  *::********:*********:**:*: ***::*** ***::. ***.:

Bol_GTR33      VNIYSGTSNFGTIVAAFLCDSYFGRYKTLSVAMIACFLGSVAMDLTAVINQLHPAKCAKE   127
AT_GTR3        VNIYGGTSNFGTIVAAFLCDSYFGRYKTLSFAMIACFLGSVAMDLTAVIHPLHPAQCAKE   125
Bol_GTR31      VNIYGGTSNFGTIVAAFLCDSYFGRYKTLSFAMIACFLGSVAMDLTAVINQLHPAKCGKE   124
Bol_GTR32      VNIYGGTSNFGTIVAAFLCDSYFGRYKTLSFAMIACFLGSVAMDLTAVINQLHPAKCAKE   143
Bol_GTR23      INAFAGTVNFGTFVAAFLCDTYFGRYKTLTVAVLACFLGSLAILLTAAVPQLHPAPCGTA   147
AT_GTR2        INAFSGTINFGTFVAAFLCDTYFGRYKTLSVAVIACFLGSFVILLTAAVPQLHPAACGTA   149
Bol_GTR21      INAFSGTINFGTFVAAFLCDTYFGRYKTLTVAVIACFLGSLVILLTAAVPQLHPAPCGTA   147
Bol_GTR22      INAFSGTINFGTFVAAFLCDTYFGRYKTLTVAVIACFLGSLVILLTAAVPQLHPAPCGTA   147
AT_GTR1        INAFSGTINFGTFIAAFLCDTYFGRYKTLSVAVIACFLGSFVILLTAAIPSLHPVACGNK   167
Bol_GTR11      INAFSGTINFGTFLAAFLCDTYFGRYKTLSVAVIACFLGSLVILLTAAVPGLHPIPCGTQ   168
Bol_GTR12      INAFSGTINFGTFFAAFLCDTYFGRYKTLSVAVIACFLGSVFILMTAAVPGLHPTPCGTK   172
               :* :.** ****:.******:********:.*::******. : :**.:  ***  *..

Bol_GTR33      IGSVCKGPSIVQIMFLAGAMVLLVIGAGGIRPCNLPFGADQFDPKTKEGKQGIESFFNWY   187
AT_GTR3        IGSVCNGPSIGQIMFLAGAMVLLVIGAGGIRPCNLPFGADQFDPKTKEGKRGIESFFNWY   185
Bol_GTR31      IGSVCKGPSIGQIMFLAGAMVLLVIGAGGIRPCNLPFGADQFNPKTKEGKRGVDSFFNWY   184
Bol_GTR32      LGTVCKGPSIGQIMFLAGAMVLLVIGAGGIRPCNLPFGADQFDPKTKEGKRGIESFFNWY   203
Bol_GTR23      --SSCSGASGGQIAFLLMGLGILVVGAGGIRPCNLAFGADQFNPKSGSGKRGIDSFFNWY   205
AT_GTR2        ADSICNGPSGGQIAFLLMGLGFLVVGAGGIRPCNLAFGADQFNPKSESGKRGIDSFFNWY   209
Bol_GTR21      L--TCTGPSGGQIAFLLMGLAFLVVGAGGIRPCNLAFGADQFNPKSESGKRGIDSFFNWY   205
Bol_GTR22      L--SCIGPSGGQIAFLLLGLGFLVVGAGGIRPCNLAFGADQFNPKSESGKRGIDSFFNWY   205
AT_GTR1        --ISCEGPSVGQILFLLMGLGFLVVGAGGIRPCNLAFGADQFNPKSESGKKGINSFFNWY   225
Bol_GTR11      --SSCQGPSEGQIAFLLMGLALLVVGAGGIRPCNLAFGADQFNPKSESGKKGINSFFNWY   226
Bol_GTR12      --SSCQGPNGGQVLFLLLGLALLVVGAGGIRPCNLAFGADQFNPKSESGKKGINSFFNWY   230
                   * * .  *: **  .: :**:********** ******:**: .**:*::******
```

```
Bol_GTR33      FFTFTFAQMVSLTVIVYVQSNVSWSIGLAIPAILMFLGCLIFFSGSKLYVKVKPSGSPIH    247
AT_GTR3        FFTFTFAQMVSLTLIVYVQSNVSWSIGLAIPAILMLLGCIIFFAGSKLYVKVKASGSPIH    245
Bol_GTR31      FFTFTFAQMVSLTLIVYVQSNVSWSIGLAIPAILMLLGCIIFFAGSKLYVKVKASGSPIH    244
Bol_GTR32      FFTFTFAQMVSLTLIVYVQSNVSWSIGLAIPAILMLLGCIIFFAGSKLYVKVKASGSPIH    263
Bol_GTR23      FFTFTFAQILSLTVVVYVQSNVSWTIGLTIPVVLMFLASVIFFAGAKLYVKIEASGSPLA    265
AT_GTR2        FFTFTFAQILSLTLVVYVQSNVSWTIGLTIPAVLMFLACLIFFAGDKLYVKIKASGSPLA    269
Bol_GTR21      FFTFTFAQILSLTLVVYIQSNVSWTIGLTIPVVLMFLACVIFFAGDKLYVKIKASGSPLA    265
Bol_GTR22      FFTFTFAQILSLTLIVYIQSNVSWTIGLTIPVVLMFLACVIFFAGDKLYVKIKASGSPLA    265
AT_GTR1        FFTFTFAQIISLTAVVYIQSNVSWTIGLIIPVALMFLACVIFFAGDRLYVKVKASGSPLA    285
Bol_GTR11      FFTFTFAQIISLTLVVYIQSNVSWTIGLSIPVGLMFLACVIFFAGHKLYVKVKASGSPLA    286
Bol_GTR12      FFTFTFAQIISLTLVVYIQSNVSWTIGLSIPVGLMFLACVIYFAGHNLYVKVKASGSPLA    290
               ********::*** :**:******:*** **. **:*..:*:*:* .****:: ****:

Bol_GTR33      SITRVIVVAIKKRKLNLVGS----MYTHTAKDFRNSKLSHTEQFRFLDKAAIQTPEDKLN    303
AT_GTR3        SITRVIVVAIKKRRLKPVGP--NELYNYIASDFKNSKLGHTEQFRFLDKSAIQTQDDKLN    303
Bol_GTR31      SITRVIVVAVKKRRLKLIGSSSEGLYNYIAKDFKNSKLSHTNQFRFLDKAAIQTPDDKLN    304
Bol_GTR32      SITRVIVVAIKKRRLKLVGSSSEGLYNYIAKDFKNSKLSHTKQFRCLDKAAIQTPDDKLN    323
Bol_GTR23      SIAHVITAAIKKHGLKSVEQPWLNLYNYYPSNYANAKLKYTEQFRFLDKAAILTPEDKLD    325
AT_GTR2        GIAQVIAVAIKKRGLKPAKQPWLNLYNYYPPKYANSKLKYTDQFRFLDKAAILTPEDKLQ    329
Bol_GTR21      SIAQVITVAIKKRGLKAVKQPWVNLYNYYPLNYANSKLKYTDQFRFLDKAAILAPEDKLE    325
Bol_GTR22      GIAQVIAVAIKKRGLKPTKEPWLNLYNYYPPKYANSKLKYTDQFRFLDKAAILAPEDKLE    325
AT_GTR1        GIARVIAAAIKKRGLKPVKQPWVNLYNHIPSNYANTTLKYTDQFRFLDKAAIMTPEEKLN    345
Bol_GTR11      GIGHVIAAAIKKRGLKPVKQPWIDLYNHIPPNYANSTLKYTDQLRFLDKAAIMTPEDKLN    346
Bol_GTR12      GIARVIAAAIKKRGLKPVKKPCSDLYNHIPPNYANSTLKYSDQFRFLDKAAVTTPDDKLK    350
               .* :**..*:**: *:        :*.:   .: *:.* ::.*:* ***:*: : ::**.

Bol_GTR33      IDGSPADPWKLCSMQQVEEVKCVIRVLPVWLSAALFYVAYIQQTTYTIFQSLQSDRHLGS    363
AT_GTR3        KDGSPVDAWKLCSMQQVEEVKCVIRVLPVWLSAALFYLAYIQQTTYTIFQSLQSDRRLGP    363
Bol_GTR31      IDGSPADLWKLCSVQQVEEVKCVIRVLPVWLSAALFYLAYIQQTTYTIFQSLQSDRRLGS    364
Bol_GTR32      IDGSPANPWNLCSLQQVEEVKCVIRVLPVWLSAALFYLAYIQQTTYTIFQSLQSDRRLGS    383
Bol_GTR23      ADGKPVNPWNLCTMQQVEEVKCIMRVLPIWFASSIYYLTIAQQMTYPVFQALQSDRRLGS    385
AT_GTR2        PDGKPADPWKLCTMQQVEEVKCIVRVLPIWFASSIYYLTITQQMTYPVFQALQSDRRLGS    389
Bol_GTR21      ADGKPTDPWKLCTMQQVEEVKCIVRVLPIWFASSIYYLTITQQMTYPVFQALQSDRRLGS    385
Bol_GTR22      ADGKPADPWKLCTMQQVEEVKCIVRVLPIWFAASIYYLTITQQMTYPVFQALQSDRRLGS    385
AT_GTR1        SDGTASDPWKLCTLQQVEEVKCIVRVIPIWFASTIYYLAITIQMTYPVFQALQSDRRLGS    405
Bol_GTR11      SDGTASDPWKLCTMQKVEEVKCILRVIPIWFACAIYYLAISMQMTYPVFQALQSDRLLGS    406
Bol_GTR12      PDGTASDPWNLCTMQQVEEVKCIVRVIPIWFACAVYYLAITLQMTYPVFQALQSDRRLGS    410
                **.  : *:**::*:******::**:*:*::.:::*::   * ** :**:***** **

Bol_GTR33      KSFQIPPATYTVFLMLGMTIFIPIYDRVLVPFLRKYTGRDSGITQLQRVGAGMFLCITSM    423
AT_GTR3        GSFQIPAGSYTVFLMLGMTIFIPIYDRVLVPFLRKYTGRDGGITQLQRVGAGLFLCITSM    423
Bol_GTR31      GTFQIPAGSYMVFLMLGMTIFIPIYDRVLVPFLRKYTGRDGGITQLQRVGAGLFLCITSM    424
Bol_GTR32      GSFKIPAATYTVFLMLGMTIFIPIYDRVLVPFLRKYTGRDGGITQLQRVGAGLFLCITSM    443
Bol_GTR23      GGFMIPAATYVVFLMTGMTLFIIFYDRVFVPTLKRITGIETGITLLQRIGAGIFFALLSL    445
AT_GTR2        GGFVIPAATYVVFLMTGMTVFIVVYDRVLVPTMRRITGLDTGITLLQRIGTGIFFATASL    449
Bol_GTR21      GGFVIPAATYVVFLMTGMTVFIIIYDRVLVPTLRRITGIDTGITLLQRIGTGIFFAFLSL    445
Bol_GTR22      RGFVIPAATYVVFLMTGMTVFIIFYDRVLVPTLRRITGIDTGITLLQRIGTGIFFAFVSL    445
AT_GTR1        GGFRIPAATYVVFLMTGMTVFIIFYDRVLVPSLRRVTGLETGISLLQRIGAGFTFAIMSL    465
Bol_GTR11      GGFKIPAATYVVFLMSGMTVFIIIYDRVLVPSLKRVTGLDTGITLLQRIGAGIFFAVLSL    466
Bol_GTR12      GGFKIPGGTYVVFLMSGMTVFIIFYDRVLVPSLRRVTGLDNGITLLQRIGSGIFFALLSM    470
                 * ** .:* **** ***:** .****:** ::: ** : **: ***:*:*: :.  *:

Bol_GTR33      MVSAIVEQRRRTVALTRPPLGFALRKGAISSMSGMWLIPQLVLMGVGDALAGVGQMEFYY    483
```

```
AT_GTR3         MVSAIVEQYRRKVALTKPTLGLAPRKGAISSMSGMWLIPQLVLMGIADALAGVGQMEFYY     483
Bol_GTR31       LVSAAVEQHRRTVALTRPTLGVAPRKGAISSMSGMWLIPQLLLMGIGDALAGVGQMEFYY     484
Bol_GTR32       MVSAAVEQHRRNVALTRPPLGFAPRKGAISSMSGMWLIPQLVLMGIADALAGVGQMEFYY     503
Bol_GTR23       IVSGFVEERRRTLALTKPTLGLAPRKGEISSMSAMWLIPQLVLAGIADAFGAIGQMEFYY     505
AT_GTR2         VVAGFVEERRRTFALTKPTLGMAPRKGEISSMSAMWLIPQLSLAGVAEAFAAIGQMEFYY     509
Bol_GTR21       VVSGFVEERRRTFALTKPTLGMAPRKGEISSMSAMWLIPQLTLAGVAEAFGAIGQMEFYY     505
Bol_GTR22       IVSGFVEERRRTFALTKPTLGMAPRKGEISSMSAMWLIPQLALAGIAEAFGAIGQMEFYY     505
AT_GTR1         LVSGFIEERRRNFALTKPTLGMAPRTGEISSMSALWLIPQLTLAGIAEAFAAIGQMEFYY     525
Bol_GTR11       LVSGFIEERRRTFALTRPTLGSEPRTGEISSMSAMWLIPQLVLAGVAEAFVAIGQMEFYY     526
Bol_GTR12       LISGFVEERRRAIALTKPTLGIEPRAGEISSMSAMWLIPQLVLAGIAEAFAAVGQMEFYY     530
                :::. :*: ** .***:* **   * * *****.:****** * *:.:*: .:*******

Bol_GTR33       KQFPENMRSFAGSLYYCGIGLASYLSSFLLSAVHNITEGSLGGNWLPEDLNKGRLEYFYY     543
AT_GTR3         KQFPENMRSFAGSLYYCGIGLASYLSTFLLSAVHDTTEGFSGGSWLPEDLNKGRLEYFYF     543
Bol_GTR31       KQFPENMRSFAGSLYYCGIGLASYLSSFLLSAVHDTTKGSLAGNWLPEDLNKGRLEYFYY     544
Bol_GTR32       KQFPENMRSFAGSLYYCGIGLASYLSSFLLSAVHDVTEGSSGGNWLPEDLNKGRLEYFYY     563
Bol_GTR23       KQFPENMRSFAGSIFYVGAGVSSYISSFLITTVHRMTQNSAGGNWLAEDLNKGRLDYFYF     565
AT_GTR2         KQFPENMRSFAGSIFYVGGGVSSYLGSFLIATVHRTTQNSSGGNWLAEDLNKGRLDLFYF     569
Bol_GTR21       KQFPENMRSFAGSIFYVGGGVSSYLGSFLIATVHRTTQNSAGGNWLAEDLNKGRLDLFYF     565
Bol_GTR22       KQFPENMRSFAGSIFYVGAGVSSYLGSFLIAMVHRTTQNSAGGNWLAEDLNKGRLDYFYF     565
AT_GTR1         KQFPENMKSFAGSIFYVGAGVSSYLASFLISTVHRTTAHSPSGNWLAEDLNKAKLDYFYF     585
Bol_GTR11       KQFPENMKSFAGSIFYVGGGVSSYLASFLISTVHRTTEHSPTGNWLAEDLNKAKLDYFYF     586
Bol_GTR12       KQFPENMKSFAGSIFYVGAGVSSYLASFLISTVHKTTEHSRSGNWLAEDLNKGKLDYFYF     590
                *******:*****::* * *::**:.:**:: **  *     *.** *****.:*: **:

Bol_GTR33       FVAGMMTINFAYFLLVSHWYRYKDIVAKDNDIDKVSV--------------      580
AT_GTR3         LVAGMMTLNLAYFLLVSHWYRYKDVVAKDKDMDKTSAEFDKV-------SV      587
Bol_GTR31       FVAGMMTLNFAYFLLVSRWYRYKDV-AKDNDMDKSSNEVDKV-------SV      587
Bol_GTR32       FVAGMMTLNFAYFLLVSHWYRYKDVVVKDKDMDKSCDEFDKV-------SV      607
Bol_GTR23       LLAGILAVNFAYFLVMARWYRYKGSDDEETSY-ETSGDIIKEQDKNKV---      612
AT_GTR2         MIAGILAVNFAYFLVMSRWYRYKGSDDEVTTY-ETNENIIKQQDKNVA---      616
Bol_GTR21       MIAGILAVNFAYFLVMSRWYRYKGSDDEVTAY-ETNGDVIKQQDKNKV---      612
Bol_GTR22       MIAGILAVNFAYFLVMSRWYRYKGSDDEVTTY-ETNGDAIKQQDKNTV---      612
AT_GTR1         MLTGLMVVNMAYFLLMARWYRYKGGNDEDITEIETNEEETKQQQLQDKNSV      636
Bol_GTR11       MLTGLMFVNFLYFLLMARWYRYKGTHDEVSYVVDTVDEETKQQDKNSV---      634
Bol_GTR12       MITGLMFVNMGYFLLMAKFYRYKVTNDEANSVIKTNEEETKENQQQDKSYV      641
                :::*:: :*: ***:::::****    :     .
```
