# Supplementary material for: Beyond defense: GTR-mediated uptake of glucosinolates drives growth and structural reprogramming in broccoli seedlings
Source: Plant Physiol. 2026 Jul 30;201(4):kiag556. doi: 10.1093/plphys/kiag556 (PMC13464505; doi:10.1093/plphys/kiag556)
Supplement: Supplementary file 1 [file kiag556_supplementary_data.zip › Supplementary_rev1.docx]

**Figure S1. Chemical structures of glucosinolates (GSLs) and fluorescent conjugates.** General core structure of GSLs (A), aliphatic GSL (B), indolic GSL (C), and aromatic GSL (D), and aromatic species conjugated to the NBD fluorophore (p-OHB-NBD) (D). The common chemical core of the aromatic GSL is highlighted in red, and the structure of the nitrobenzoxadiazole (NBD) fluorophore is highlighted in green.

**Figure S2.** Phylogenetic analysis of glucosinolate transporters (GTRs) in Brassica oleracea var. italica (broccoli) and Arabidopsis thaliana. Sequence alignment was performed with Clustal Omega, and the phylogenetic tree was constructed using the PhyML method. Species names were abbreviated as follows: Bol (broccoli), At (A. thaliana).

**Figure S3.** **Functional characterization of broccoli GTRs in a heterologous system**. Uptake of (A) 3-But, (B) 4-MTB, (C) 4-MSB, (D) 5-MSP, (E) I3M, (F) NMOI3M, (G) 2-PE and (H) pOH by AtGTR1, GTR1.2, GTR2.2, GTR2.3, GTR3.3 and water-injected MOCK oocytes expressed in Xenopus laevis, analysed by LC–MS. Yellow bars indicate aliphatic GSLs, purple bars indicate indolic GSLs and blue bars indicate aromatic GSLs. Each bar represents the mean ± SE (n = 5). Significant differences between GTR-expressing oocytes and the MOCK control were determined by t-tests (* p < 0.05, ** p < 0.01, *** p < 0.001). Different letters indicate significant differences according to one-way ANOVA (p < 0.05) followed by Tukey’s post hoc test.

**Figure S4. Negative controls for the root internalization assay of fluorescent GSL.** (A, B) Root autofluorescence.(C, D) Root of broccoli seedlings treated with 50 µM of the fluorophore NBD. (E, F) Root of broccoli seedlings treated with 25 µM of the fluorophore NBD. (G–I) Root of broccoli seedlings treated with 50 µM NBD and 40 µM propidium iodide (PI)

**Figure S5. Differentially expressed genes (DEGs) in 7-day-old broccoli seedlings treated with GSLs compared with untreated controls.** Principal component analysis (PCA) of RNA-seq data from biological replicates of control (CA, CR) and GSL-treated (GA, GR) samples in the aerial part (A) and roots (C). Volcano plots show DEGs identified in the aerial part (B) and roots (D). The x-axis shows the Log₂ fold change, while the y-axis represents statistical significance (–Log₁₀ p-value). Each point corresponds to an individual gene: yellow indicates genes with p < 0.05, purple highlights genes with p < 0.05 and |fold change| > 1, and grey denotes genes without significant differential expression.

**Figure S6. Cell wall remodelling-related genes.** Heatmap of significantly regulated genes associated with cell wall structural remodeling and vesicular trafficking in broccoli roots. Yellow indicates low expression and purple indicates high expression (Z-score). For each gene, the log fold change (logFC), mean expression (AveExpr), and gene name are shown. Significant differences between GSL-treated and control plants were determined using Student’s t-tests: *p < 0.05, **p < 0.01, ***p < 0.001.
